# Development and Evaluation of a New Spectral Disease Index to Detect Wheat Fusarium Head Blight Using Hyperspectral Imaging

**DOI:** 10.3390/s20082260

**Published:** 2020-04-16

**Authors:** Dongyan Zhang, Qian Wang, Fenfang Lin, Xun Yin, Chunyan Gu, Hongbo Qiao

**Affiliations:** 1 National Engineering Research Center for Agro-Ecological Big Data Analysis & Application, Anhui University, Hefei 230601, China; zhangdy@ahu.edu.cn (D.Z.); p18201091@stu.ahu.edu.cn (Q.W.); linfenfang@126.com (F.L.); p17301129@stu.ahu.edu.cn (X.Y.); 2 School of Geography and Remote Sensing, Nanjing University of Information Science & Technology, Nanjing 210044, China; 3 Institute of Plant Protection and Agro-Products Safety, Anhui Academy of Agricultural Sciences, Hefei 230031, China; guchunyan@aaas.org.cn; 4 School of Information and Management Science, Henan Agricultural University, Zhengzhou 450002, China

**Keywords:** hyperspectral imaging, spectral indices, random forest, growth stage, Fusarium head blight

## Abstract

Fusarium head blight (FHB) is a major disease threatening worldwide wheat production. FHB is a short cycle disease and is highly destructive under conducive environments. To provide technical support for the rapid detection of the FHB disease, we proposed to develop a new Fusarium disease index (FDI) based on the spectral data of 374–1050 nm. This study was conducted through the analysis of reflectance spectral data of healthy and diseased wheat ears at the flowering and filling stages by hyperspectral imaging technology and the random forest method. The characteristic wavelengths selected were 570 nm and 678 nm for the late flowering stage, 565 nm and 661 nm for the early filling stage, 560 nm and 663 nm for the combined stage (combining both flowering and filling stages) by random forest. FDI at each stage was derived from the wavebands of each corresponding stage. Compared with other 16 existing spectral indices, FDI demonstrated a stronger ability to determine the severity of the FHB disease. Its determination coefficients (*R*^2^) values exceeded 0.90 and the *RMSEs* were less than 0.08 in the models for each stage. Furthermore, the model for the combined stage performed better when used at single growth stage, but its effect was weaker than that of the models for the two individual growth stages. Therefore, using FDI can provide a new tool to detect the FHB disease at different growth stages in wheat.

## 1. Introduction

The production of wheat plays important social and economic roles, and the quality and safety issues related to these functions have been the focus of research at the national level and abroad. Fusarium head blight (FHB) is a wheat disease caused by the fungus *Gibberella zeae* (*Fusarium graminearum*) and often severely affects wheat yield and quality. Wheat infected with FHB accumulates a large amount of toxins in its grains, thereby seriously threatening public health. These bacterial toxins can contaminate flour and persist in the food chain for long periods, producing carcinogens. Therefore, FHB has become one of the crop diseases of great concern worldwide [1,2].

Conventional crop disease detection methods range from the naked eye to random monitoring, which have the disadvantages of strong subjectivity, high labor intensity, and time consumption. With the rapid development of spectral technology, hyperspectral imaging has been gradually applied to non-destructive detection of plant diseases and insect pests [3]. Hyperspectral images can provide hundreds of thousands of continuous narrow band data points and are very sensitive to changes in the physical and chemical parameters of plants caused by disease infection. These changes have gradually developed into effective features for expressing plant growth information and have proven to be effective in identifying plant diseases and insect pests [4]. Zheng et al. [5] used wavelengths of 570 nm, 525 nm, 705 nm, 860 nm, 790 nm, and 750 nm to identify yellow rust successfully in the early and middle stages of wheat growth. Huang [6] and others, based on the Relief-F algorithm, proposed that 515 nm, 698 nm, and 738 nm are key wavelengths for distinguishing wheat powdery mildew from other diseases. Bauriegel [7] believe that 550–560 nm and 665–675 nm are the best bands for field identification of FHB. However, with the increase in spectral and image spatial resolution, the simultaneous increase of data dimensions, noise, and redundant spectra pose considerable challenges to data storage, processing, and analysis [8].

The vegetation index is an effective method often used in the field of optical remote sensing to reflect changes in plant physiological and biochemical parameters. This index is a simple and efficient spectral data processing method that combines a few characteristic bands in a certain mathematical form. This method greatly eliminates the redundancy of hyperspectral data, has a small amount of calculation, and is widely used to estimate crop yields [9], pigment content [10], canopy structure [11], and changes in water status [12]. In recent years, exclusive spectral indexes have been proposed and demonstrated unique advantages in plant disease detection. Zhang et al. [8] developed a hyperspectral index based on hyperspectral microscopic images to identify FHB ears with classification accuracy of 0.898. Devadas et al. [13] observed that healthy and susceptible (yellow rust, leaf rust, stem rust) wheat can be distinguished based on the anthocyanin reflectance index. Rumpf et al. [14] combined the spectral index and support vector machine to identify beet leaf spot, leaf rust, and powdery mildew at an early stage, and the classification accuracy was above 0.65. The results of these studies indicate that a spectral index calculated by spectral reflectance at a special wavelength position has high potential for applications in the fields of crop diseases and insect pests. However, these proposed spectral indexes do not clearly indicate the applicable growth stage or only consider a certain growth period of the crop.

The pathological characteristics of wheat after being infected with FHB differ at separate stages, which may cause inconsistent relationships between the spectral index and the status of FHB during different growth periods. In many studies on spectral indexes, researchers have usually pooled observation data at different stages of the entire growth stage to explore characteristic bands and construct spectral indices, thereby weakening the inconsistencies of FHB status in different growth stages [15]. FHB usually occurs during the flowering and filling stages of wheat. At maturity, the damage caused by FHB to wheat yield and quality has been determined. Currently, conducting research on FHB identification is of minimal importance. Therefore, this study focuses on the accuracy and stability of the disease severity monitoring model for FHB at late flowering and early filling stages, with the goal of providing assistance for the scientific control of the disease. The main research objectives are as follows: (1) Based on the difference in spectral responses between healthy and infected ears, the most suitable characteristic wavelengths for identifying FHB were selected and determined by the random forest (RF) technique in the late flowering stage and early filling stage. (2) A Fusarium disease index (FDI) was constructed in the form of the normalized wavelength difference and compared with classical disease index to evaluate the accuracy and stability of FDI.

## 2. Materials and Methods

### 2.1. Wheat Material

**Figure 1:**
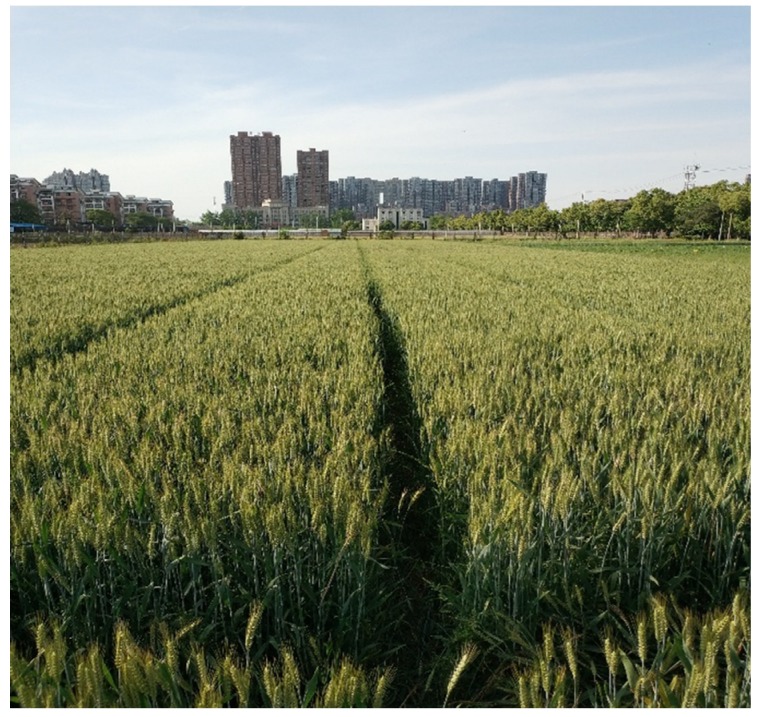
Experimental field plots.

This study was carried out at the experimental base of the Anhui Academy of Agricultural Sciences (31°89′ N, 117°1′ E) in China from 2017 to 2018 (Figure 1). The tested wheat variety was Xinong 979, which is moderately susceptible to FHB. A 10 × 10 m experimental plot was divided into an inoculation area (50 m^2^) and a control area (50 m^2^). In the early flowering stage, a small sprayer was used to spray a freshly prepared spore suspension (*F. graminearum*) on the ears of wheat in the inoculation area. The control area was sprayed with pesticide (Carbendazim, 750 g/hm^2^) once between the full heading stage and the early flowering stage (18 April 2018) to prevent FHB and ensure a sufficient number of healthy samples for comparative research. Other field management techniques such as fertilization and irrigation were carried out in the two experimental plots according to local agronomic measures. In this study, 149 and 229 wheat ears were collected at the late flowering (3 May 2018) and early filling (9 May 2018) stages for a total of 378 samples.

### 2.2. Inoculum Production

Under the bench with a sterile environment, the infected wheat grains were treated twice with mercury dichloride–alcohol–sterilized water. The treated grains were added to potato dextrose agar medium, and the culture was grown at 25 °C for three days. Five mycelium plugs were picked at the edge of the colony and placed in 100 mL carboxymethyl cellulose medium for four days. The conidia were filtered with two pieces of filter paper and centrifuged at 5000 rpm for 5 min. The concentration of the spore suspension was adjusted to 1 × 10^5^/mL with sterile water.

### 2.3. Data Acquisition and Processing

#### 2.3.1. Spectral Measurements

**Figure 2:**
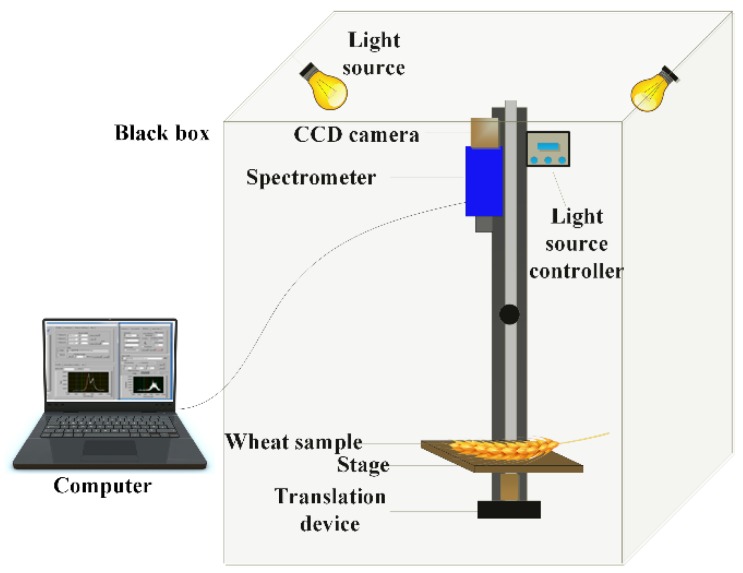
Hyperspectral imaging system.

The spectral reflectance of the ears was measured using an SOC710E spectrometer (Surface Optics Corporation, San Diego, CA, USA). The spectral range of this instrument is 374–1050 nm, and the spectral resolution is 2.3 nm. After picking wheat ears in the field, the samples were quickly sent to the laboratory in a fresh-keeping box, and the spectral data were collected in a dark room. Wheat ears were placed on a black platform, and the exposure time and the distance between the platform and the lens were adjusted so that the wheat ears could be clearly imaged. Two 75-Watt halogen lamps were placed on both sides of the dark room to illuminate the sample. The hyperspectral imaging system is shown in Figure 2. Measurements on the whiteboard (with a reflectance of approximately one) and dark current (with a reflectance of approximately zero) were performed for spectral correction. The reflectance value of the dark current was recorded by covering the lens with a black cloth. The correction formula is as follows:

(1) $$R=\frac{R_{original}-R_{dark}}{R_{white}-R_{dark}}$$

where *R*_original_ represents the original spectral reflectance, *R*_dark_ is the reflectance value of the dark current, *R*_white_ is the reflectance value of the reference whiteboard, and R is the corrected spectral reflectance of the image.

#### 2.3.2. Calculation of Disease Severity

**Figure 3:**
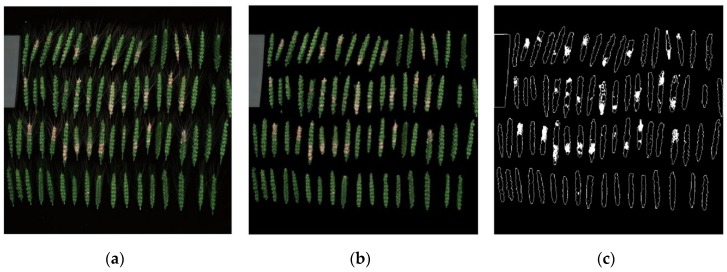
Extraction of diseased spots from wheat ears, (**a**) original image; (**b**) image of wheat tip and stalk removal; (**c**) image of diseased spots extraction.

With respect to ear scale, the disease severity is mainly quantified by the ratio of the diseased area on the ear to the whole ear area. Therefore, Fusarium head blight lesions were segmented from the whole ear to measure relative lesion area on ears. First, the third channel image of the original red green blue (RGB) image (Figure 3a) was processed with binarization and morphological corrosion and expansion to remove the tip of wheat and stalks in the image (Figure 3b).

Because the three components in the RGB image were represented by a three-dimensional Cartesian coordinate system, they were highly correlated and relatively heterogeneous, resulting in a small difference between the healthy area and the diseased area that was difficult to segment. The color space of YDbDr was used to separate the brightness and color difference, which was more suitable for distinguishing between green and red yellow susceptible areas. Therefore, the RGB image after the wheat tip and stalk removal was transferred to the YDbDr color space, and a threshold segmentation method was adopted to extract the ear disease spots (Figure 3c).

The severity of the FHB is expressed by the ratio of the number of pixels in the disease spot region to the pixel number in the whole wheat ear region, as shown in Equation (2):

(2) $$SI=\frac{S_{lesion}}{S_{all}}$$

*SI* represents the severity of FHB, *S*_lesion_ is the number of pixels in the disease spot area, and *S*_all_ is the number of pixels in the whole wheat ear region.

#### 2.3.3. Characteristic Band Selection

The RF algorithm was used to select the characteristic wavelengths that are sensitive to FHB. This algorithm is an ensemble learning algorithm based on multiple classification and regression trees (CARTs) proposed by Breiman [16] and is often used for characteristic wavelength selection in hyperspectral data analysis [17,18]. In this algorithm, the bootstrap resampling method is used to generate the training set; attributes are measured according to the minimum Gini index principle, and CART is gradually established. Subsequently, the classification of samples is determined by combining the voting of each decision tree. At the same time, the samples that do not appear in the training set are designated as “bag data” and are used to predict the accuracy of the algorithm.

The Gini index is an attribute splitting method based on impurity. The smaller the impurity, the worse the dispersion degree of the variables and the more information that is obtained [19]. The formula for calculating the impurity Gini index **G** is shown in Equation (3):

(3) $$G(a)=1-\sum_{i=1}^{c}P_{i}^{2}$$

where c is the number of sample categories, and P_i_ is the probability that the sample corresponding to an attribute a belongs to category c_i_ (c_i_ represents the i-th category).

Because the Gini impurity index is negatively related to the available information, this study used the Gini purity index to convert the purity and available useful information into a positive correlation to more intuitively reflect the impact of features on the classification effect. The calculation formula is as follows:

(4) $$G_{purity}(a)=\sum_{i=1}^{c}P_{i}^{2}$$

Through the converted formula, the Gini purity index of characteristic f can be obtained as follows:

(5) $$G(f)=\sum_{i=1}^{k}\frac{n_{i}}{N}G_{purity}(a_{i})$$

where N is the number of samples, k is the number of categories of a certain attribute a, a_i_ is a certain category of attributes, and n_i_ is the number of samples corresponding to a certain category. The greater the purity of a feature, the stronger the ability of the feature to recognize the sample. The calculation formula for the importance measurement of the feature is as follows:

(6) $$S(v)=\frac{1}{t}\sum_{u=1}^{t}G(f_{\mathrm{uv}})$$

where t is the number of training datasets in the RF, G(*f*_uv_) is the purity of the v-th dimension eigenvector in the u-th training dataset (v = 1, 2, 3, ...., k), and k is the overall dimension of the sample. Finally, the required characteristic wavelengths were obtained according to the positive maximum value and the negative minimum value of the importance score.

### 2.4. Construction of Proposed New Spectral Disease Index for Indentifing Wheat FHB

Previous studies [6,20] have shown that the disease spectral index in the form of the normalized wavelength difference is very sensitive to spectral changes caused by powdery mildew, stripe rust, and aphids. Therefore, this study used the normalized wavelength difference in combination with characteristic wavelengths to construct the exclusive FDI for each period. The calculation is carried out via Equation (7):

(7) $$\mathrm{FDI}=\frac{R_{\lambda 1}-R_{\lambda 2}}{R_{\lambda 1}+R_{\lambda 2}}$$

where *R_λ_*_1_ represents the reflectance at the *λ*1 wavelength and *R_λ_*_2_ represents the reflectance at the *λ*2 wavelength.

### 2.5. Traditional Spectral Indices for Wheat FHB Detection

Pigment content can provide information about the physiological state of leaves; consequently, a spectral index that can characterize the plant pigment content is highly related to plants’ physiological and biochemical changes and is often used for non-destructive detection of plant diseases and insect pests. Sixteen commonly used spectral indexes (Table 1) were selected and compared with the FDI proposed in this study to evaluate FDI’s ability to identify and distinguish infected ears.

### 2.6. Linear Regression Model and Validation

A linear regression model was used to model the relationship between spectral indices (FDI and existing spectral indices) and the severity index (SI) at different growth stages. The evaluation indexes of the model included the root mean square error (RMSE) and the coefficient of determination (*R*^2^). RMSE represents the standard deviation of the difference between the predicted value and the measured value. *R*^2^ used to measure the proportion of variation in the dependent variable that can be explained by the independent variable. The closer *R*^2^ is to 1, the closer the regression line is to each observation point, and the better the regression fit.

To make the distribution of samples more uniform, the SI values of the samples were arranged in descending order and then divided into a training dataset and test dataset in a 3:1 proportion. Specifically, one sample was taken from a group of four samples as the test dataset, and the remaining three were used as the training dataset. In the model, using FDI as the independent variable and SI as the dependent variable, the relationship between FDI and SI in different periods was determined by regression analysis. In a single growing period, the FDI of the sample in the training set was calculated, and the linear regression equation between it and the corresponding SI was established to obtain the *R*^2^ and RMSE in the training dataset. The SI of each sample in the test dataset was predicted by a linear regression equation and FDI, and the *R*^2^ and RMSE of the prediction set were obtained by comparing the actual SI with the predicted SI. In addition, the samples in the combined stage were modeled and predicted, and the linear regression equation of the combined stage was used to predict the test dataset samples of the late flowering and early filling stages.

## 3. Results and Analysis

### 3.1. Spectral Response Differences of Wheat FHB with Different Disease Severities

**Figure 4:**
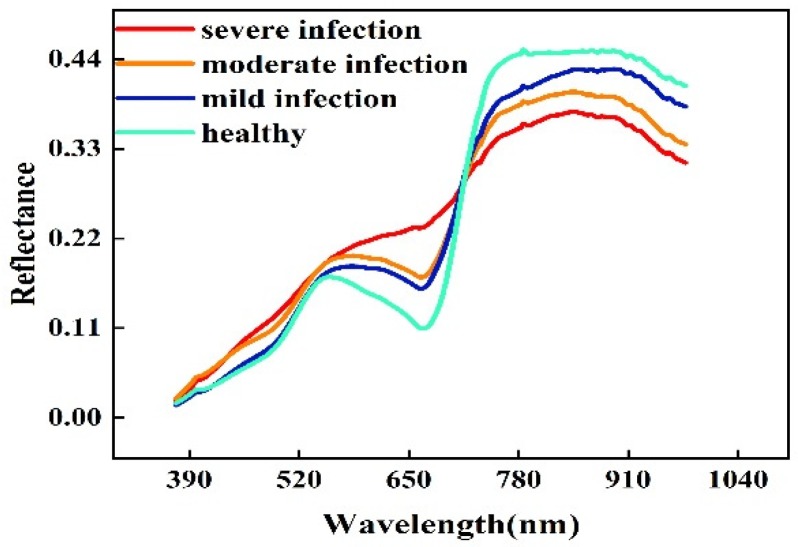
Spectral reflectance curves of wheat ears with different disease severities.

In the process of using hyperspectral data to accurately identify diseases, the study of spectral signatures under different disease severities is the basis for screening and identifying sensitive bands of diseases. Figure 4 shows the spectral signature of wheat ears with different infection levels. Generally, in the range of the 550–720 nm band, the spectral reflectance of healthy ears is lower than that of infected samples, with an obvious green peak and red valley; accordingly, these two spectral features disappear in the severely infected ears. Conversely, in the range of the 721–1000 nm band, the more severely infected the sample, the lower its reflectivity. The difference in the responses of wheat ears with different severities in the 550–720 nm and 721–1000 nm bands may be related to the difference in the pigment content and moisture content in mesophyll tissue [9]. Furthermore, with the increase in the severity of wheat diseases, a clear red edge moved in the short-wave direction. The above obvious spectral signature differences provide an important optical basis for analyzing and constructing the relationship between the spectral index and FHB severity in this study.

### 3.2. Construction of Proposed New Spectral Disease Index for Identifying Wheat FHB 

#### 3.2.1. Characteristic Bands for Identifying FHB at Different Growth Stages

**Figure 5:**
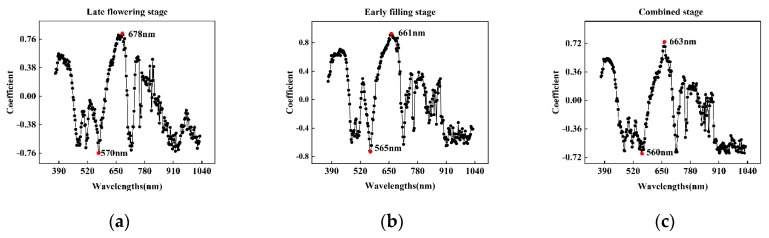
Weight coefficients calculated by RF at the late flowering stage (**a**), early filling stage (**b**) and combined stage (**c**).

RF was used to select characteristic wavelengths in samples during the late flowering stage, early filling stage, and combination of both. The weight coefficients of all wavelengths were calculated in the spectral range of 374–1050 nm. Except for the extreme points, the weight coefficients of adjacent wavelengths were similar, thus indicating that the information of adjacent wavelengths was highly correlated. To reduce the redundant information and maximize the effective spectral information, this study selected the wavelength corresponding to the positive highest weight coefficient and the negative lowest weight coefficient as the characteristic wavelengths. As shown in Figure 5, the characteristic wavelengths were 570 nm and 678 nm at the late flowering stage, 565 nm and 661 nm at the early filling stage, and 560 nm and 663 nm at the combined stage.

Characteristic wavelengths selected from the different stages were all in the range of 565–680 nm. Combined with Figure 5, this band range shows a significant difference between healthy and infected wheat ears. Furthermore, the characteristic wavelengths selected in the two growth stages were different, from 678 nm and 570 nm in the late flowering stage to 661 nm and 565 nm in the early filling stage. With the development of FHB, the position of the characteristic wavelength moved in the direction of the short wave, which was consistent with the spectral change of the previously reported disease stress plants; specifically, the red edge shifted in the direction of the blue wave. Notably, the characteristic wavelengths of the combined stage are closer to those of the early filling period, which may be due to the sample size in the early filling stage (229) being larger than that in the late flowering stage (149); moreover, the incidence characteristics of FHB were obvious in this growth period.

#### 3.2.2. Construction of New Fusarium Disease Index for Identifying Wheat FHB

**Figure 6:**
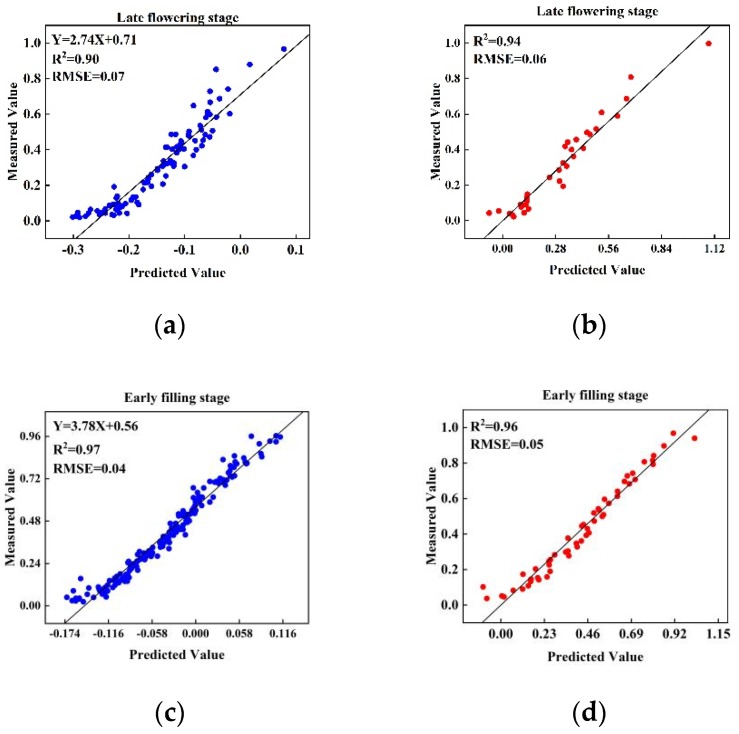

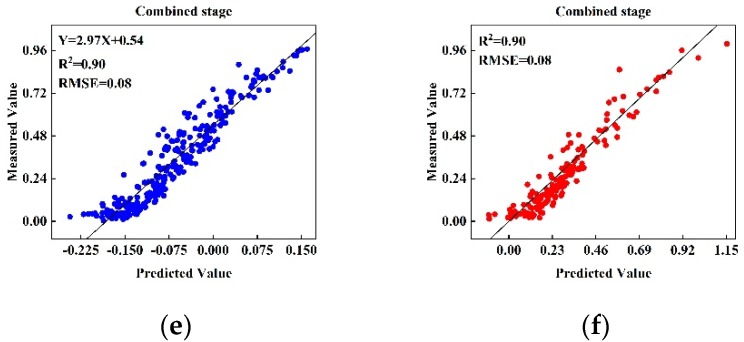
Evaluation of regression models in the training and test datasets at the late flowering stage (**a**,**b**), the early filling stage (**c**,**d**) and the combined stage (**e**,**f**).

In this study, with FDI as the independent variable and SI as the dependent variable, the relationship between FDI and SI in different stages was evaluated by linear regression analysis (Figure 6). FDI made an accurate prediction of the SI of wheat ears at the late flowering stage, early filling stage, and combined stage (*R*^2^ was greater than 0.90, RMSE was less than 0.08). At each stage, the *R*^2^ and RMSE of the training and test datasets were close, indicating that the model had a strong generalization ability. From the results of the training and test datasets, the FDI prediction was the most accurate in the early filling stage, followed by the late flowering period, and the lowest in the combined stage. (0.96, 0.94, and 0.90, respectively).

**Figure 7:**
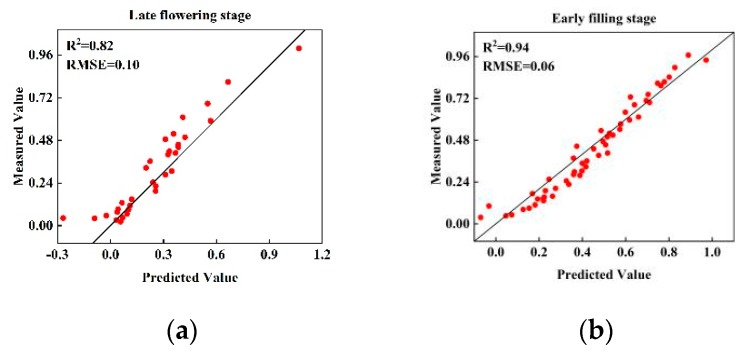
Evaluation of regression models at the combined stage used at the late flowering stage (**a**) and early filling stage (**b**).

In this study, the regression model obtained from the combined stage was applied to the test set of the late flowering and early filling stages (Figure 7). The results obtained by applying the regression model established through the combined stage to the test datasets of the late flowering and early filling stages (*R*^2^ = 0.91 and 0.94, respectively) were slightly lower than those of the regression models of the late flowering and early filling stages (*R*^2^ = 0.94 and 0.96, respectively), especially in the late flowering stage.

### 3.3. Comparison of FDI and Traditional Spectral Indices

To verify the application potential of the FDI for detection of FHB, the results were compared with 16 other published spectral indexes at different stages (Table 2, Table 3 and Table 4). In the late flowering stage and combined stage, only the FDI proposed in this study had an *R*^2^ above 0.9 in the training and the test datasets. In the early filling stage, only FDI and NRI had an *R*^2^ above 0.9 in both the training and test datasets. The characteristic wavelengths of FDI (661 nm, 565 nm) and NRI (670 nm, 570 nm) were also close. The prediction results of FDI were higher than those of other spectral indexes in different stages, especially in the late flowering stage, which indicated that the FDI had excellent monitoring accuracy in the early stage of FHB infection. Furthermore, the detection capabilities of the selected spectral indices were different at separate stages, but the *R*^2^ of FDI at every stage was greater than 0.9. Among these indices, nitrogen reflectance index (NRI), transformed vegetation index (TVI), and green index (GI) performed relatively well at different growth stages. What they all have in common is that they have better predictions during a single growth period than during the combined growth period. The diversity of samples in the combined stage may increase the difficulty of model prediction in this period because the characteristic wavelengths are dynamically changing in different growth stages [5]. At the same time, the performance of some indices in different growth stages will be very different. For example, modified chlorophyll absorption in the reflectance index (MCARI) has much better predictive power in the early filling period (*R*^2^ = 0.67) than in the late flowering period (*R*^2^ = 0.41); normalized pigment chlorophyll ratio index (NPCI) has much better predictive power in the combined period (*R*^2^ = 0.77) than in the early filling period (*R*^2^ = 0.17). The same index responds differently to diseases in different growth stages, which may be affected by the pathogenic mechanism of vegetation [13].

## 4. Discussion

### 4.1. Analysis of Spectral Characteristics for Identifying Wheat FHB

Previous studies have demonstrated that changes in crop physiological and biochemical parameters lead to changes in spectral reflectance, which is the basis for optical technology used to diagnose the severity of FHB [5]. As the symptoms of FHB are different in separate growth stages of wheat, characteristic wavelengths used to identify the severity of FHB are also different, so it is necessary to extract these wavelengths for different stages. In addition, there are some differences in the spectra of wheat ears with different degrees of infection in specific bands. Figure 4 shows the destruction of chloroplasts in the ear tissue of FHB which causes the chlorophyll in the cells to continuously degrade, and the reflectivity of the spectrum in the chlorophyll band (560–675 nm and 682–733 nm) decreases rapidly. At the same time, the decrease of the chlorophyll content in these cells reduces the possibility of photon reemission and reabsorption in this wavelength range, resulting in an increase in spectral reflectivity and the blue shift of the “red edge”. These changes become more obvious with the increase in the degree of infection. The characteristic wavelengths selected in this study are in the range of 560–680 nm, including the green reflection peak and red absorption valley, and can characterize the characteristics of wheat ears. According to the characteristic wavelengths of the late flowering stage (570 nm and 678 nm) and early filling stage (565 nm and 661 nm), the 570 nm and 565 nm wavelengths are near the green peak, while 678 nm and 661 nm are near the red valley. Therefore, the characteristic wavelengths selected in this study are key wavelengths for identifying wheat ears with FHB. Bauriegel [7] also confirmed the importance of this band in the early detection of wheat ear scabs.

### 4.2. Comparison of Application Effects between Proposed New FDI and Traditional Spectral Indices

The most common and serious symptom of wheat FHB, ear rot, often begins at the early flowering stage. Therefore, the monitoring of wheat FHB at the flowering stage is highly valuable. However, the FHB fungi in the flowering stage are in the stage of mass reproduction, and the physiological and biochemical characteristics of the infected ears are not obvious, which makes it more difficult for a conventional spectral index to detect the severity of FHB at the flowering stage. The 16 published spectral indices selected in this study were constructed by collecting sample data from different stages of multiple growth stages, so their accuracies in detecting the severity of FHB at the late flowering stage were relatively poor. In this study, the sample models of the late flowering stage and combined stage were applied to the test set of the late flowering stage. The results show that the model obtained from the late flowering stage sample was more suitable for the detection of FHB at the late flowering stage than the model obtained from the combined stage sample. The characteristic wavelength selected from the samples at the late flowering stage (570 nm and 678 nm) was therefore more suitable for FHB detection than the characteristic wavelength selected during the combined stage (560 nm and 663 nm).

In the sample verification of the combined stage, the accuracies of the 16 published spectral indexes were not satisfactory because none of them were able to achieve an *R*^2^ above 0.85 in both the training and test datasets. These spectral indices are mainly based on the leaves or canopies of crops rich in chlorophyll, and few diagnostic studies have used these indices to evaluate the severity of FHB in wheat ears, which is a special part with a low chlorophyll content. In the early filling period, in addition to the FDI, the NRI, plant senescence reflectance index (PSRI), GI, normalized difference vegetation (NDVI), and optimized soil-adjusted vegetation index (OSAVI) also had accurate detection results. This may be because the morphology and cell structure of wheat ears caused by FHB are more obvious than those at the late flowering, so other spectral indices may be more accurate in this context. Notably, among these spectral indexes, NRI and GI both performed strongly at the late flowering and early filling stages but performed relatively poorly in the combined stage. This shows that the diversity of samples has an important effect on predictions of FHB severity, which demonstrates the importance of subdividing the growth period when exploring the forecasts of FHB severity.

### 4.3. Analysis of Other Influential Factors

Hyperspectral data contain hundreds of narrow-band data points, but the adjacent wavelength information is often highly correlated, so the use of full-band information will only increase the complexity of data acquisition and calculation [5]. Usually, the most effective information is only contained in some specific bands, and the rest is redundant information [36]. In addition, the high price of hyperspectral imaging systems will also limit the application potential of the technology. This study explored the characteristic wavelengths at different stages to design a multispectral camera with a low price, a fast processing speed, and wide applications for specific identifications of FHB in different growth stages. In this study, the *R*^2^ of predicted SI and FDI exceeded 0.90 in the late flowering, early filling, and combined stages. Considering the influence of man-made or natural environmental factors, this prediction accuracy is acceptable.

In addition, the spatial distribution and severity of FHB diseases are greatly affected by the genetic resistance of different varieties as well as environmental and agricultural management factors. Therefore, the factors that affect the biophysical and biochemical parameters of plants will affect the identification of FHB. This study was conducted under laboratory conditions, and its direct application in the field requires further verification. In the future, the growth stage will be further subdivided, especially in the early stages of FHB occurrence, such as mid-flowering, late flowering, early filling, and mid-filling, to achieve early detection, protection, and evaluation.

## 5. Conclusions

Monitoring wheat infection by FHB at different growth stages is important in making a decision on the use of pesticides to protect wheat from FHB and to evaluate yield losses. In this study, RF was used to select characteristic wavelengths for the late flowering stage, early filling stage, and combined stage of both. These wavebands were 570 nm and 678 nm for the late flowering stage, 565 nm and 661nm for the early filling stage, 560 nm and 663 nm for the combined stage. In the light of above wavelengths, FDI at each stage were constructed for establishing linear regression models with SI. Every model showed a high predictive accuracy with the test datasets, with their *R^2^* values exceeding 0.90. In addition, the *R^2^* of the model established at the late flowering stage and early filling stage was better than that of the combined stage, and the *R^2^* of applying the model of the combined stage to the test dataset at the late flowering stage and filling stage also decreased. Therefore, it is indicated that FHB shows different spectral characteristics at each growth stage, which provides a favorable basis for detecting the severity of the FHB disease at different growth stages in the future. However, additional studies are needed to verify the universality of FDI on different wheat varieties and in different field experiment settings.

## Figures and Tables

**Table 1:** Traditional spectral indices tested in the study.

Full Name of Spectral Index	Spectral Index Abbreviation	Calculation Formula
nitrogen reflectance index [21]	NRI	$\left(R_{570}-R_{670})/(R_{570}+R_{670}\right)$
photochemical reflectance index [22]	PRI	$\left(R_{531}-R_{570})/(R_{531}+R_{570}\right)$
transformed vegetation index [23]	TVI	$0.5\times [120\times (R_{750}-R_{550})-200\times (R_{750}+R_{550})]$
transformed chlorophyll absorption in the reflectance index [24]	TCARI	$3\times [(R_{700}-R_{670})-0.2\times (R_{700}-R_{550})\times (R_{700}/R_{670})]$
modified chlorophyll absorption in the reflectance index [25]	MCARI	$\left[(R_{700}-R_{670})-0.2\times (R_{700}-R_{550})]\times R_{700}/R_{670}\right)$
red-edge vegetation stress index [26]	RVSI	$[(R_{712}+R_{752})/2]-R_{732}$
plant senescence reflectance index [27]	PSRI	$(R_{678}-R_{500})/R_{750}$
green index [28]	GI	$R_{554}/R_{677}$
structural independent pigment index [29]	SIPI	$\left(R_{800}-R_{445})/(R_{800}+R_{680}\right)$
normalized pigment chlorophyll ratio index [30]	NPCI	$\left(R_{680}-R_{430})/(R_{680}+R_{430}\right)$
normalized difference vegetation index [31]	NDVI	$\left(R_{840}-R_{675})/(R_{840}+R_{675}\right)$
optimized soil-adjusted vegetation index [32]	OSAVI	$1.16\times [(R_{800}-R_{670})/(R_{800}+R_{670}+0.16)]$
Lichtenthaler’s indices [33]	Lic1	$\left(R_{800}-R_{680})/(R_{800}+R_{680}\right)$
Lichtenthaler’s indices [34]	Lic2	$R_{400}/R_{690}$
anthocyanin reflectance index [35]	ARI	${\left(R_{550}\right)}^{-1}-{\left(R_{700}\right)}^{-1}$
physiological reflectance index [22]	PHRI	$\left(R_{550}-R_{531})/(R_{550}+R_{531}\right)$

**Table 2:** Comparison of proposed FDI and traditional spectral indices at the late flowering stage.

Spectral Indices	Late Flowering Stage				
	Regression Equation	Training Set		Test Set	
		*R* ^2^	RMSE	*R* ^2^	RMSE
NRI	y = −2.77x + 0.68	0.87	0.08	0.86	0.09
PRI	y = −5.68x + 0.02	0.08	0.22	−7.9	0.21
TVI	y = −0.06x + 1.36	0.86	0.09	0.91	0.07
TCARI	y = −4.01x + 1.13	0.75	0.11	0.78	0.11
MCARI	y = −21.73x + 1.27	0.38	0.18	0.41	0.19
RVSI	y = 6.97x + 0.48	0.13	0.21	−4.46	0.22
PSRI	y = 4.96x + 0.01	0.73	0.12	0.79	0.12
GI	y = −0.85x + 1.48	0.86	0.09	0.88	0.07
SIPI	y = −3.39x + 2.40	0.74	0.12	0.68	0.12
NPCI	y = 2.58x − 0.61	0.41	0.18	−0.1	0.18
NDVI	y = −2.34x + 1.52	0.82	0.1	0.86	0.08
OSAVI	y = −2.79x + 1.55	0.81	0.1	0.81	0.1
Lic1	y = −2.40x + 1.52	0.82	0.1	0.86	0.08
Lic2	y = −0.81x + 0.63	0.02	0.23	−34.83	0.24
ARI	y = 0.36x − 0.12	0.2	0.21	−2.24	0.21
PHRI	y = −17.22x + 1.04	0.4	0.18	−1.82	0.23
FDI	y = 2.74x + 0.17	0.90	0.07	0.94	0.06

**Table 3:** Comparison of proposed FDI and traditional spectral indices at the early filling stage.

Spectral Indices	Early Filling Stage				
	Regression Equation	Training Set		Test Set	
		*R* ^2^	RMSE	*R* ^2^	RMSE
NRI	y = −3.2x + 0.55	0.93	0.07	0.92	0.07
PRI	y = −9.52x − 0.30	0.19	0.23	−2.14	0.23
TVI	y = −0.05x + 1.02	0.89	0.08	0.89	0.08
TCARI	y = −3.35x + 1.02	0.87	0.09	0.85	0.10
MCARI	y = −16.72x + 1.14	0.76	0.13	0.67	0.14
RVSI	y = 5.09x + 0.47	0.07	0.25	−14.73	0.26
PSRI	y = 3.60x − 0.15	0.88	0.09	0.85	0.10
GI	y = −1.25x + 1.79	0.89	0.09	0.85	0.09
SIPI	y = −3.74x + 2.44	0.78	0.12	0.71	0.12
NPCI	y = 3.48x − 1.16	0.52	0.18	0.17	0.19
NDVI	y = −2.45x + 1.32	0.87	0.09	0.85	0.10
OSAVI	y = −2.74x + 1.32	0.89	0.09	0.87	0.09
Lic1	y = −2.45x + 1.32	0.87	0.09	0.85	0.10
Lic2	y = −2.44x + 1.28	0.09	0.25	−6.40	0.24
ARI	y = 0.39x − 0.18	0.17	0.23	−4.87	0.25
PHRI	y = −13.41x + 1.13	0.17	0.23	−4.19	0.25
FDI	y = 3.78x + 0.56	0.97	0.04	0.96	0.05

**Table 4:** Comparison of FDI and traditional spectral indices at the combined stage.

Spectral Indices	Combined Stage				
	Regression Equation	Training Set		Test Set	
		*R* ^2^	RMSE	*R* ^2^	RMSE
NRI	y = −2.69x + 0.56	0.82	0.11	0.78	0.11
PRI	y = −7.56x − 0.16	0.46	0.19	0.03	0.18
TVI	y = −0.05x + 1.05	0.73	0.13	0.64	0.13
TCARI	y = −4.02x + 1.13	0.81	0.11	0.75	0.13
MCARI	y = −20.80x + 1.29	0.63	0.16	0.48	0.18
RVSI	y = 5.75x + 0.45	0.11	0.24	−8.03	0.26
PSRI	y = 2.57x − 0.05	0.72	0.14	0.63	0.14
GI	y = −0.97x + 1.53	0.79	0.12	0.73	0.12
SIPI	y = −3.71x + 2.51	0.34	0.21	−1.03	0.22
NPCI	y = 1.67x − 0.36	0.79	0.12	0.77	0.12
NDVI	y = −2.24x + 1.31	0.66	0.15	0.51	0.15
OSAVI	y = −2.54x + 1.32	0.66	0.15	0.56	0.14
Lic1	y = −2.24x + 1.32	0.66	0.15	0.51	0.15
Lic2	y = −2.45x + 1.27	0.73	0.13	0.70	0.13
ARI	y = 0.56x − 0.43	0.69	0.14	0.49	0.16
PHRI	y = −6.66x + 0.01	0.08	0.25	−9.57	0.24
FDI	y = 2.97x + 0.54	0.90	0.08	0.90	0.08
